# Pretreatment Glasgow prognostic score as a predictor of outcomes in nivolumab-treated patients with advanced gastric cancer

**DOI:** 10.1371/journal.pone.0247645

**Published:** 2021-02-26

**Authors:** Nagahiro Tokuyama, Naoki Takegawa, Michiko Nishikawa, Aya Sakai, Takuya Mimura, Saeko Kushida, Hidetaka Tsumura, Yoshinobu Yamamoto, Ikuya Miki, Masahiro Tsuda

**Affiliations:** Department of Gastroenterology, Hyogo Cancer Center, Akashi, Hyogo, Japan; Chang Gung Memorial Hospital at Linkou, TAIWAN

## Abstract

In Japan, South Korea, and Taiwan, nivolumab might provide overall survival benefits for patients with advanced gastric cancer. However, it is effective only in a limited number of patients. The Glasgow prognostic score is an indicator of the systematic inflammatory response and nutritional status. This study aimed to investigate the ability of the Glasgow prognostic score and other markers to predict the outcomes of patients treated with nivolumab. We reviewed the medical records of patients treated for advanced gastric cancer and who received nivolumab between February 2015 and June 2019 at Hyogo Cancer Center. The patients were categorized into two groups according to their Glasgow prognostic scores. Overall, 53.3% and 46.7% of the patients were assigned to groups with Glasgow prognostic scores of 0 and 1/2, respectively. The median durations of progression-free and overall survival of the participants were 2.3 and 5.7 months, respectively. The patients with a Glasgow prognostic score of 0 had significantly higher median overall survival than those with scores of 1 or 2 (16.4 vs. 4.2 months; *p* = 0.0006). This observation suggests that a pretreatment Glasgow prognostic score of 0 is associated with better outcomes, and this scoring system may be used as a predictor of outcomes in patients with advanced gastric cancer treated with nivolumab.

## Introduction

Although the prognosis for advanced gastric cancer (AGC) remains poor, overall survival (OS) has increased following improvements in systemic chemotherapy treatment [1–3]. In a phase III study [4], nivolumab, which blocks programmed cell death 1, has shown a survival benefit over placebo in patients who were previously treated for AGC regardless of human epidermal growth factor receptor 2 (HER2) status [5]. Consequently, nivolumab has been approved as a standard monotherapy in Japan, South Korea, and Taiwan. However, at present, the number of patients with gastric cancer who benefit from the therapeutic effects of nivolumab is limited. Whereas the Tumor Proportion Score is correlated with treatment outcomes in non-small cell lung cancer patients treated with nivolumab [6], this score does not predict the effect of nivolumab treatment in AGC. The Combined Positive Score, mismatch repair status, and Epstein-Barr virus positivity are also potential markers for the efficacy of pembrolizumab and nivolumab treatments [7,8]; however, additional studies are needed. Accurate prediction contributes to better management of patients with AGC; therefore, it is extremely important to identify clinically practical markers to predict the outcome of nivolumab treatment in patients with AGC.

The Glasgow prognostic score (GPS) has been extensively investigated for its ability to predict the postoperative outcomes of various cancers [9–11]. The GPS is a combination of serum C-reactive protein (CRP) and albumin levels, which are biomarkers of the systematic inflammatory response and nutritional status, respectively [12]. Previous studies have indicated that a high GPS is a negative prognosticator in gastric cancer patients undergoing gastrectomy [13–16]. However, the use of the GPS for predicting treatment outcomes in patients with AGC who were treated with nivolumab has not been assessed. Therefore, in the present study, we analyzed the predictive ability of GPS and other markers in nivolumab-treated patients with AGC.

## Materials and methods

### Patients

**Fig 1:**
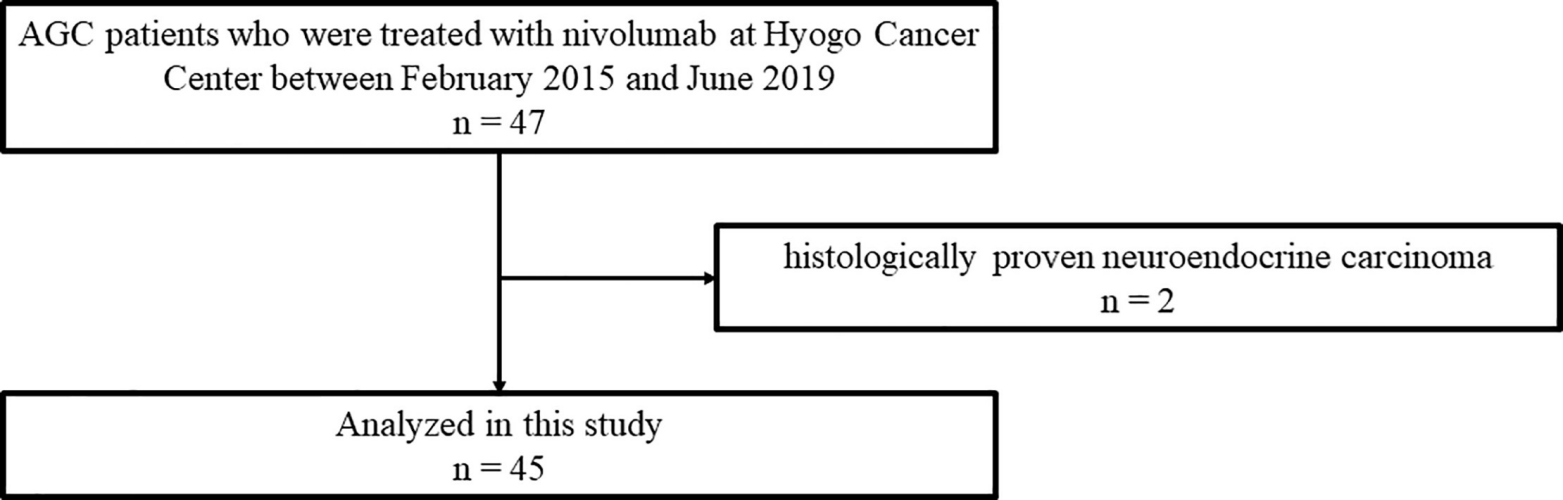
Consort flow diagram.

We retrospectively reviewed the medical records of all patients with advanced or recurrent (stage IV) gastric cancer who had been treated with nivolumab at Hyogo Cancer Center between February 2015 and June 2019. In total, 47 patients were administered nivolumab during this period. The eligibility criteria for the present study were the following: 20 years of age or older; histologically proven adenocarcinoma of the stomach or esophagogastric junction; measurable or evaluable lesions according to the Response Evaluation Criteria in Solid Tumors (RECIST) version 1.1; preserved Eastern Cooperative Oncology Group (ECOG) performance status (PS) of ≤ 2; no major organ dysfunction; no active multiple primary cancers; available data regarding serum CRP and albumin levels (determined within 7 days before the initiation of nivolumab treatment); refractoriness or intolerance to fluoropyrimidines (fluorouracil, S-1, or capecitabine) in a first-line setting (includes 4 patients who did not receive platinum); and refractoriness or intolerance to taxanes (paclitaxel, docetaxel, or nab-paclitaxel) or irinotecan in a second-line setting. This study was approved by the ethics committee of Hyogo Cancer Center (Approval number G-53, 2019). Although the records are not anonymous, the ethics committee waived the requirement for informed consent for this study. Two patients were excluded from our analysis because they had histologically proven neuroendocrine carcinoma. A total of 45 patients with AGC met the criteria and were included in this study (Fig 1).

### Treatment

Nivolumab was intravenously administered at a dose of 3 mg/kg every 2 weeks except that the treatment regime was adjusted to 240 mg/body per 2 weeks after September 2018. In total, 34 and 8 patients were administered the dose of 3 mg/kg and 240 mg/body, respectively. The other three patients initially received nivolumab at 3 mg/kg and then received 240 mg/body after September 2018. The treatment was repeated until disease progression, unacceptable toxicity, or refusal by the patient to continue. The end of the follow-up period was June 2019.

### Evaluation

Information for each patient was retrospectively evaluated using electronic medical records. Items surveyed in this study included age, sex, ECOG PS, HER2 status, tumor status, site of the primary tumor, metastatic sites, liver metastasis, peritoneal metastasis, ascites, number of previous therapies, Lauren classification, neutrophil-to-lymphocyte ratio (NLR), and the GPS. Progression-free survival (PFS) was defined as the time from the first administration of nivolumab to the first occurrence of disease progression or death. OS was defined as the time from the onset of nivolumab treatment until death by any cause. Information regarding chemotherapy included the administration doses, best response, PFS, and OS. The overall response rate was assessed according to RECIST version 1.1[17].

### Statistical analyses

For each patient, a GPS was calculated from CRP and albumin levels obtained within 7 days prior to the initiation of nivolumab treatment. Each patient with increased CRP (> 1.0 mg/dL) and hypoalbuminemia (< 3.5 g/dL) levels was assigned a score of 2. Patients with only one abnormal value were assigned a score of 1. Patients who had CRP and albumin levels within normal ranges were assigned a score of 0. We sorted the patients into two groups according to their GPSs, GPS-H (1 or 2) and GPS-L (0). In addition, we obtained NLRs by dividing absolute neutrophil counts by absolute lymphocyte counts. We used an NLR threshold of 5, consistent with previous studies [18,19]. Kaplan-Meier analyses of PFS and OS were performed based on these cut-off values, with differences between each pair of groups assessed with the log-rank test. The hazard ratio and 95% confidence interval were calculated using the univariable Cox proportional hazards model. Parameters with *p* < 0.05 in the univariable analysis were selected as covariates in the multivariable Cox proportional hazards regression analysis. Relationships between the best response and biomarker categories were analyzed with Fisher’s exact test. All *p* values were based on a two-sided hypothesis, with those < 0.05 considered statistically significant. Statistical analyses were performed with JMP software version 8.00 (SAS Institute).

## Results

### Patient characteristics

A total of 45 patients were included in this study. The clinical characteristics of these patients are summarized in Table 1. The information was obtained at the onset of nivolumab treatment. All the patients received nivolumab as a third-line or later treatment. The median age of the patients was 65 years (range 40–81 years). Fourteen females and 31 males were included. Computed tomography (CT) or magnetic resonance imaging was generally performed every 6–12 weeks. One patient, who was enrolled in a clinical study and allocated to a nivolumab treatment group, was evaluated by CT examination every 6 weeks initially; after the end of cycle 9, CT examination was performed every 12 weeks, according to the study protocol. All the other patients were evaluated every 6–9 weeks.

**Table 1:** Patient characteristics.

Characteristic	*N* = 45
Median age (range) (years)	65 (40–81)
Sex (male/female)	31/14
ECOG PS (0/1/2)	16/23/6
HER2 status (+/–/unknown)	10/33/2
Tumor status (unresectable/recurrence)	27/18
Site of primary tumor (U/M/L)	18/32/30
Metastatic sites (1/2/≥ 3)	19/19/7
Liver metastasis (+/–)	10/35
Peritoneal metastasis (+/–)	26/19
Ascites (no/low/medium/massive)	20/12/5/8
Number of previous therapies (2/≥ 3)	21/24
NLR (< 5/≥ 5)	41/4
GPS (0/1/2)	25/18/2

ECOG PS, Eastern Cooperative Oncology Group performance status; HER2, Human Epidermal Growth Factor Receptor 2; U/M/L, upper (U), middle (M), and lower (L) sections of the stomach; low ascites, localized buildup of ascites in the perihepatic or pelvic space; medium ascites, ascites of intermediate size; massive ascites, accumulation of ascites extending throughout the abdominal cavity; NLR, neutrophil-to-lymphocyte ratio; GPS, Glasgow prognostic score.

### Univariate and multivariable analysis of markers for survival outcome

**Fig 2:**
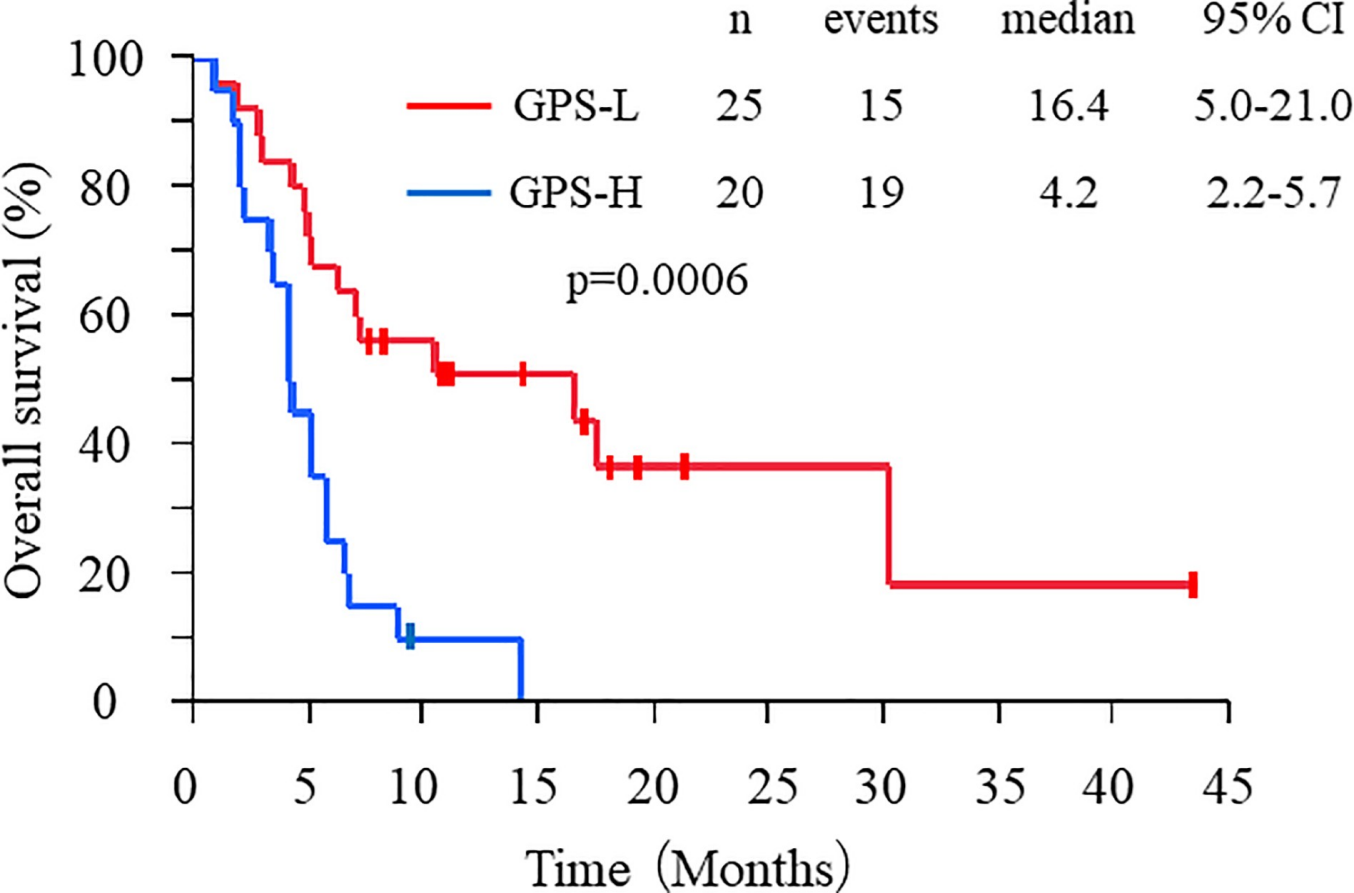
OS of patients sorted by GPS. Kaplan–Meier analysis of OS in GPS-L (red line) and GPS-H (blue line) patients.

At the time of the data cut-off, 41 patients (91%) had progressed and 34 (76%) had died. The median PFS was 2.3 months (95% confidence interval: 1.4–3.5 months), and the median OS was 5.7 months (95% confidence interval: 4.2–8.2 months). According to the Kaplan–Meier analysis, patients with GPS-L experienced significantly longer OS than those with GPS-H (16.4 vs. 4.2 months, respectively; *p* = 0.0006; Fig 2). The univariate analysis results indicated that factors significantly associated with better OS included sex (male), ECOG PS 0, no peritoneal metastasis, no ascites, high serum albumin levels, and GPS-L status (Table 2). Having a small study population rendered the multivariate analysis unreliable, where no ascites, low serum albumin (not high), and GPS-L status were significantly associated with better OS (S1 Table).

**Table 2:** Univariate analysis of OS.

		OS		
Parameter	Category	HR	95% CI	*p*-value
Age	< 65/≥ 65	1.65	0.83–3.43	0.16
Sex	Male/female	2.22	1.06–4.49	0.035
ECOG PS	0/1, 2	3.32	1.53–8.01	0.002
Lauren classification	Intestinal/diffuse	1.37	0.70–2.78	0.36
HER2	+/–	1.80	0.78–4.86	0.18
Tumor status	Unresectable/recurrent	0.97	0.47–1.95	0.94
Liver metastasis	+/–	1.02	0.47–2.56	0.96
Metastatic sites	1/≥ 2	1.78	0.88–3.77	0.109
Peritoneal metastasis	+/–	0.29	0.13–0.61	0.0009
Ascites	+/–	0.26	0.12–0.55	0.0003
Number of previous therapies	2/≥ 3	0.85	0.43–1.71	0.65
Serum albumin	≥ 3.5/< 3.5 (g/dL)	2.72	1.28–5.76	0.0061
Serum CRP	≤ 1.0/> 1.0 (mg/dL)	2.09	0.83–4.64	0.079
NLR	< 5/≥ 5	1.54	0.37–4.39	0.51
GPS	0/1, 2	3.63	1.73–7.91	0.0006

OS, overall survival: HR, hazard ratio; CI, confidence interval; ECOG PS, Eastern Cooperative Oncology Group performance status; HER2, Human Epidermal Growth Factor Receptor 2; CRP, C-reactive protein; NLR, neutrophil-to-lymphocyte ratio; GPS, Glasgow prognostic score.

### Response to treatment according to GPS

Overall response was evaluated in 22 patients with measurable lesions. Partial response was observed in 7 patients (31.8%), and disease control was observed in 13 patients (59.1%). When stratified according to GPS-L and GPS-H, the GPS-L patients tended to correlate with better response rates (42.9% vs. 12.5%; *p* = 0.14; Table 3).

**Table 3:** Response rates according to GPS.

GPS	All	Responders	Non-responders	ORR	*p*-value
GPS-L	14 (63.6%)	6	8	42.9	0.14
GPS-H	8 (36.3%)	1	7	12.5	

GPS, Glasgow prognostic score; GPS-L, GPS 0; GPS-H, GPS 1 or 2; ORR, overall response rate.

## Discussion

Several studies have reported biomarkers that can predict the outcome of nivolumab treatment for AGC [20,21]. To the best of our knowledge, this is the first study to evaluate the role of the pretreatment GPS as a predictor of the outcome in nivolumab-treated patients with AGC. We found that GPS-L was associated with better outcomes, suggesting that GPS status can be used as a predictor to identify patients who are likely to experience a favorable clinical outcome.

In this study, patients treated with nivolumab monotherapy had median PFS and OS of 2.3 and 5.7 months, respectively, which are better outcomes than those reported in a previous study [4]. However, nivolumab was administered as a fourth- or later-line treatment in 79.1% of the patients in the previous study, whereas 46.7% of our patients were administered nivolumab monotherapy as a third-line treatment. Thus, this difference may explain the observed discrepancies in clinical outcomes. Contrary to a previous report [21], the NLR was not significantly associated with OS in nivolumab-treated AGC patients in our analysis. In univariate analyses, serum albumin was also a predictive factor; additionally, we consider that the immunosuppressive tumor microenvironment (indicated by a higher serum CRP) may play a critical role in nivolumab treatment. This scenario may explain why the GPS was a more powerful predictor of outcomes than serum albumin alone (the estimated hazard ratio for OS was 2.72 for Alb < 3.5 vs. Alb > 3.5 (*p* = 0.0061) and 3.63 for GPS-H vs. GPS-L (*p* = 0.0006) in the present study, and the NLR was not.

ECOG PS, peritoneal metastasis, and ascites, which are potential prognosticator candidates described in a previous report [20], were pronounced predictive markers in our study. These observations suggest that a low GPS, a better ECOG PS, no peritoneal metastasis, and no ascites reflect a healthier systemic immune environment.

The KEYNOTE-061 trial [22], the aim of which was to compare the clinical outcomes of pembrolizumab and paclitaxel treatments in patients with second-line PD-L1-positive gastric cancer, did not reveal any superior effect of pembrolizumab over paclitaxel. In subgroup analysis, the efficacy of pembrolizumab treatment was greater in patients with PD-L1 Combined Positive Score positive status and for patients with high-level microsatellite instability in their tumors. Although these findings were consistent with a previous retrospective study involving nivolumab treatment [20], the markers utilized require additional examinations, such as immunohistochemistry or polymerase chain reaction fragment analysis [23]. Indeed, the advantages of using peripheral blood as a source of indicators over tumor tissues include both ease of access and facile assay performance. Moreover, we consider that combining several biomarkers may assist clinical decision-making regarding patients treated with nivolumab.

GPS-L may reflect the local tumor immunity available to be activated by nivolumab (which is sufficient to mediate a clinically meaningful anti-tumor effect). A retrospective study in esophageal cancer [24] has demonstrated that a better serum albumin-based nutritional condition is significantly associated with the CD8-positive cell count. A recent study has shown that higher CRP levels indicate an immunosuppressive tumor microenvironment in patients with clear cell renal cell carcinoma [25]. The authors found that CRP levels were significantly higher in patients with strong infiltration of CD8+ Foxp3+ cells, and cancer-specific survival was significantly worse in these patients than in patients with weak infiltration. As the immune response is balanced by interactions between T cells and other regulatory cells [26,27], the GPS, which is a combination of the levels of serum CRP and albumin, might enable a better prediction of nivolumab treatment outcome.

There are limitations to our study. First, we did not evaluate the outcomes of patients who had not received nivolumab. Second, we did not analyze PD-L1 expression, mismatch repair status, or cancer genome alterations in the patients, which are also reported predictive factors [20]. Third, a further prospective validation study is needed to evaluate the clinical use of our findings. Lastly, measurable tumors were more frequent among the GPS-L patients in our study, which may have affected the treatment outcome [28].

In conclusion, we demonstrated that the pretreatment GPS is a predictor of outcomes in patients with AGC at the initiation of nivolumab treatment. Although the sample size was too small to conclude that the combination of two baseline blood biomarkers has definitive application, the predictive value of this combination warrants further investigation in additional patients. As the GPS can easily be calculated using data from widely available tests without additional costs, this parameter may help identify nivolumab-treated patients with good outcomes and help optimize treatment plans for AGC.

## Supporting information

S1 Table Multivariate analysis of OS. (DOCX)
