# Supplementary material for: Pretreatment Glasgow prognostic score as a predictor of outcomes in nivolumab-treated patients with advanced gastric cancer
Source: PLoS One. 2021 Feb 26;16(2):e0247645. doi: 10.1371/journal.pone.0247645 (PMC7909621; doi:10.1371/journal.pone.0247645)
Supplement: S1 Table — (DOCX) [file pone.0247645.s001.docx]

**Table S1 Multivariate analysis of OS**

|  |  |  | OS | | |  |
| --- | --- | --- | --- | --- | --- | --- |
| Parameter | Category |  | HR | 95% CI | *p*-value | |
| Age | < 65/ > 65 |  | 1.56 | 0.74–3.42 | 0.25 | |
| Sex | Male/female |  | 2.23 | 0.95–5.39 | 0.065 | |
| ECOG PS | 0/ 1, 2 |  | 2.04 | 0.77–5.87 | 0.15 | |
| Peritoneal metastasis | +/ – |  | 0.48 | 0.11–1.83 | 0.29 | |
| Ascites | +/ – |  | 0.27 | 0.07–0.98 | 0.046 | |
| Serum albumin | > 3.5/ < 3.5 (g/dL) |  | 0.09 | 0.01–0.46 | 0.0043 | |
| GPS | 0/ 1, 2 |  | 19.21 | 4.26–102.1 | 0.0002 | |

OS, overall survival: HR, hazard ratio; CI, confidence interval; ECOG PS, Eastern Cooperative Oncology Group performance status; GPS, Glasgow prognostic score
